# Notch signaling during development requires the function of *awd*, the *Drosophila* homolog of human metastasis suppressor gene *Nm23*

**DOI:** 10.1186/1741-7007-12-12

**Published:** 2014-02-14

**Authors:** Marilena Ignesti, Marilena Barraco, Gouthami Nallamothu, Julie A Woolworth, Serena Duchi, Giuseppe Gargiulo, Valeria Cavaliere, Tien Hsu

**Affiliations:** 1 Dipartimento di Farmacia e Biotecnologie, Alma Mater Studiorum Università di Bologna, Via Selmi, 3, Bologna 40126, Italy; 2 Department of Medicine, Boston University School of Medicine, Boston, Massachusetts 02118, USA; 3 Department of Pathology and Laboratory Medicine, Medical University of South Carolina, Charleston, South Carolina 29425, USA; 4 Graduate Institute of Systems Biology and Bioinformatics, National Central University, Jhongli, Taiwan; 5 Present address: Institute of Hematology “L. e A. Seràgnoli”, University of Bologna, Bologna, Italy; 6 Present address: Bone Regeneration Laboratory, Research Institute Codivilla-Putti, Rizzoli Orthopaedic Institute, Bologna, Italy

**Keywords:** Awd, Notch signaling, Endocytosis

## Abstract

**Background:**

The *Drosophila abnormal wing discs* (*awd*) belongs to a highly conserved family of genes implicated in metastasis suppression, metabolic homeostasis and epithelial morphogenesis. The cellular function of the mammalian members of this family, the Nm23 proteins, has not yet been clearly defined. Previous *awd* genetic analyses unraveled its endocytic role that is required for proper internalization of receptors controlling different signaling pathways. In this study, we analyzed the role of Awd in controlling Notch signaling during development.

**Results:**

To study the *awd* gene function we used genetic mosaic approaches to obtain cells homozygous for a loss of function allele. In *awd* mutant follicle cells and wing disc cells, Notch accumulates in enlarged early endosomes, resulting in defective Notch signaling. Our results demonstrate that *awd* function is required before γ-secretase mediated cleavage since over-expression of the constitutively active form of the Notch receptor in *awd* mutant follicle cells allows rescue of the signaling. By using markers of different endosomal compartments we show that Notch receptor accumulates in early endosomes in *awd* mutant follicle cells. A trafficking assay in living wing discs also shows that Notch accumulates in early endosomes. Importantly, constitutively active Rab5 cannot rescue the *awd* phenotype, suggesting that *awd* is required for Rab5 function in early endosome maturation.

**Conclusions:**

In this report we demonstrate that *awd* is essential for Notch signaling via its endocytic role. In addition, we identify the endocytic step at which Awd function is required for Notch signaling and we obtain evidence indicating that Awd is necessary for Rab5 function. These findings provide new insights into the developmental and pathophysiological function of this important gene family.

## Background

The *Drosophila awd* (*abnormal wing discs*) gene was identified in a genetic screen for genes involved in imaginal disc development [1,2]. It encodes the *Drosophila* homolog of human metastasis suppressor gene *Nm23*[3,4]. The *Nm23* gene family (also termed *NME*) consists of ten related genes in mammals [5] with the *NME1* and *NME2* isoforms most implicated in tumor progression and sharing about 78% of amino acid identity with the Awd protein. During *Drosophila* development, *awd* is critical for epithelial morphogenesis [6] and has been linked to AMP kinase-regulated energy-sensing [7]. Human and murine *Nm23* has been shown in cancer cell xenografts to inhibit metastasis, but not primary tumor growth [8]. On the other hand, in other cancer cohorts, particularly those of ovarian cancers, up-regulated *Nm23* levels have been correlated with poor prognosis [9,10], suggesting an oncogenic function. These discrepancies have so far been difficult to reconcile because the exact cellular function of Nm23 has remained unclear, although several molecular activities have been assigned to the Nm23 family of proteins. Nm23 belongs to a classic nucleoside diphosphate kinase (NDPK) family that generates nucleoside triphosphates using adenosine triphosphates (ATP) as a phosphate source [11], but other activities, such as histidine-dependent protein kinase [12-14], nuclease [15-18] and lipid bilayer-binding [19,20], have also been documented. Interestingly, in *Drosophila*, *awd* has been shown to interact genetically with *dynamin* to promote endocytosis [6,21], although it is not yet clear which endocytic process is regulated by *awd*. In neurons, *awd* has been shown to promote Dynamin-mediated neurotransmitter uptake at the neuromuscular junction [22]. Proper tracheal branching morphogenesis requires *awd* function to regulate internalization and signaling of the fibroblast growth factor receptor (FGFR) encoded by the *breathless* gene [23]. During oogenesis *awd* is down-regulated in border cells to allow for accumulation of and chemotactic signaling from the platelet-derived growth factor/vascular endothelial growth factor (PDGF/VEGF) receptor (PVR) [24]. Awd also regulates Domeless signaling via modulating endocytosis [24]. Moreover, loss of *awd* function in the follicular epithelium causes mislocalization of β-catenin and DE-cadherin, resulting in over-accumulation of these adherens junction components and disruption of epithelial integrity [25]. During our analyses of *awd* function in the follicular epithelium, we also noted a proliferation abnormality in *awd* mutant cells that is reminiscent of the Notch signaling defect. This observation prompted us to revisit the original ‘abnormal wing discs’ phenotype, which led to the discovery of the classic ‘notched wing’ phenotype in flies carrying mosaic *awd* mutant clones. Notch pathway is a highly conserved cell-cell communication pathway and functions to regulate many different cellular processes during embryonic development and in adulthood [26]. Canonical Notch signaling requires binding of membrane-bound Notch receptor to membrane-bound ligand Delta/Serrate/Lag2 (DSL) on the juxtaposed cells. The interaction triggers proteolytic cleavage in the extracellular juxtamembrane region of Notch (S2 cleavage), separating the ligand-bound extracellular domain and the membrane-bound NEXT (Notch EXternal Truncation) [27]. NEXT is then subjected to intra-membrane proteolysis by γ-secretase (S3 cleavage). The proteolysis releases the intracellular domain of Notch (NICD), which translocates into the nucleus and regulates transcription of target genes by association with transcriptional cofactors of the CBF1-Su(H)-Lag1 (CSL) family [26,28-30]. More recently, it has been shown that in some cell types, Notch entry into the endocytic pathway is critical for proper Notch activation and signaling [31-34]. Since Notch signaling may function either as a tumor suppressor or as an oncogene, depending on the tissue context [35], the functional relationship between *Nm23/awd* and Notch may provide important insights into the seemingly contradictory roles of *Nm23* in tumor progression. In addition, elucidating the Notch signaling defect in *awd* mutant cells should also shed light on the *awd* action in the endocytic pathway.

In the present study, we show that *awd* function is required for proper Notch signaling in follicle cells and imaginal disc cells. Genetic studies reveal that in *awd* mutants, Notch is blocked from entry into late endosomes and accumulates in abnormal, Avalanche (Avl)-positive vesicles, precluding signal activation.

## Results

### Notch signaling requires *awd* function in follicle cells and imaginal disc cells

The *Drosophila* egg chamber consists of a 16-germ cell syncytium enveloped by a monolayer of follicular epithelium [36]. The process of proliferation and differentiation of the follicle cells is complex and under stringent control [37-39]. One critical event is the cessation of mitosis in mid-oogenesis. The proliferation of follicle cells occurs before stage 7 (up to 30 hours after the egg chamber buds off from the germarium at stage 2; total egg chamber development time from stage 2 to stage 14 is approximately 70 hours). Notch signaling that regulates cell proliferation in the follicle cells is activated at stage 6, which results in down-regulation of *cut* and *cyclin B*, among other Notch target genes, and cessation of mitosis [40-43]. From stage 7 to 10A (approximately 3 hours after stage 6) the follicle cell chromosomes continue to duplicate three times to generate polyploidity (endocycles). Disruption of Notch signaling causes extension of the proliferative program beyond stage 6 and follicle cells go through additional cell divisions without cell growth, resulting in increased cell number but reduced cell size.

**Figure 1:**
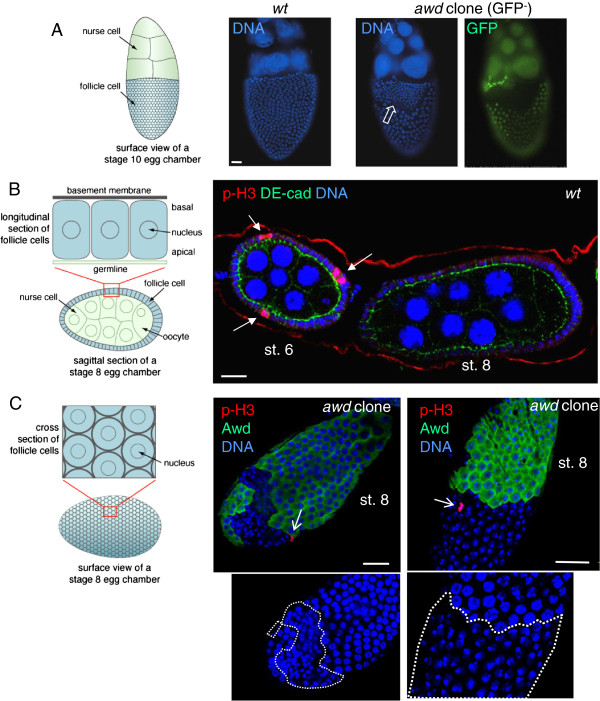
**Dysregulated proliferation in *awd *mutant follicle cells. (A-B)** Control egg chambers were dissected from *yw* (representing wild-type) females. Egg chambers containing *awd* clones (no GFP) were dissected from *hs-flp; +/+; Ubi-GFP, FRT*^*82B*^*/FRT*^*82B*^*, awd*^*j2A4*^ females. **(A)** In follicular epithelium, *awd* mutant clones show more numerous but smaller nuclei (empty block arrow) in comparison with the adjacent normal follicle cells and with *yw* egg chambers (left). To visualize nuclei the stage 10 egg chambers were stained with DAPI (blue). **(B) ***yw* egg chambers were stained for p-H3 (red), DE-cadherin (green) and DNA (blue). p-H3 detects mitotic cells only in pre-stage 7 eggs (arrows). **(C)** Egg chambers containing *awd* clones were dissected from *yw; en2.4-Gal4*^*e22c*^*, UAS-flp/+; FRT*^*82B*^*/FRT*^*82B*^*, awd*^*j2A4*^, and stained for p-H3 (red), Awd (green) and DNA (blue). In *awd* mutant clones (lack of Awd expression), p-H3 positive cells can be detected post-stage 6 (sharp arrows). Duplicate images showing only nuclear staining (insets) highlight the smaller nuclei in *awd* mutant clones (dashed lines). Schematic representations of the positioning and viewing of follicle cells in the egg chamber are shown in the left side of A, B and C. Bars are 20 μm. DAPI, 4',6-diamidino-2-phenylindole; p-H3, phosphorylated histone H3.

We have previously shown that *awd* is involved in regulating epithelial integrity of the follicle cells via its endocytic activity [25]. During the course of examining follicular function of *awd*, we also noticed that at later stages (after stage 8) the *awd* mutant clones often contain more numerous but smaller cells, suggesting faulty Notch signaling (Figure 1A). Since *awd* null alleles are lethal, the phenotypes in follicle cells, an adult tissue, are generated by mitotic recombination using the FLP/FRT system [44]. In this report, we employed different genetic methods that allow for induced mitotic recombination using temporal or tissue-specific expression of the recombinase FLP [45] or allow for co-expression of other transgenes in the *awd* mutant clones using the mosaic analysis with a repressible cell marker (MARCM) system [46]. While specific genetic strategies will be pointed out when appropriate, it is worth noting that the *Notch* phenotypes generated are consistent regardless of the FLP/FRT variations.

**Figure 2:**
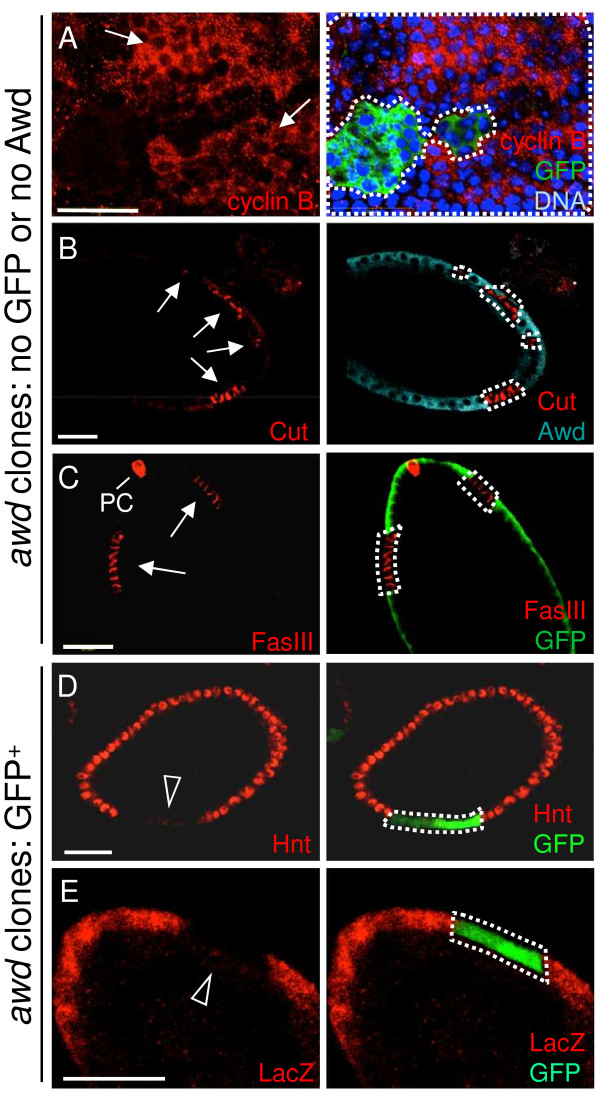
**Altered expression of Notch signaling target genes in *awd *clones. (A and C)** Stage 7–8 egg chambers were dissected from *hs-flp; +/+; Ubi-GFP, FRT*^*82B*^*/FRT*^*82B*^*, awd*^*j2A4*^ females, and the *awd* mutant clones were identified by lack of GFP staining (green). **(B, D-E)** Stage 7 egg chambers were dissected from *hs-flp/GbeSu(H)*_*m8*_*-lacZ; act-Gal4, UAS-GFP/+; FRT*^*82B*^*, act-Gal80/FRT*^*82B*^*, awd*^*j2A4*^ females. In **(A-C)** the *awd* mutant clones were identified by lack of Awd or GFP staining (green), while in **(D-E)** the *awd* mutant clones are GFP positive. In all panels, *awd* mutant clones are marked with dashed lines. **(A)** Cyclin B (red), **(B)** Cut (red) and **(C)** Fasciclin III (FasIII; in red) are normally negatively regulated by Notch signaling, but up-regulated in *awd* mutant clones (lack of GFP or Awd; arrows). **(D)** Hindsight (Hnt; in red) is normally induced by Notch signaling, and is down-regulated in *awd* clones (GFP-positive; empty arrowhead). **(E)** The *lacZ* reporter gene expression driven by Notch-activated *Su(H)* promoter [*GbeSu(H)*_*m8*_*-lacZ*] is lost in *awd* clones (GFP-positive; empty arrowhead). Bars are 20 μm.

Immunostaining with the mitotic marker phosphorylated histone H3 (p-H3) shows that *awd* mutant cells continue to divide after stage 6. In wild-type follicle cells p-H3 positive cells are detectable only up to stage 6 of oogenesis (Figure 1B). Note that p-H3 is only observed in M phase. Since mitosis of follicle cells is not synchronized, only a few cells are stained at any given time. In *awd* mutant cells p-H3 staining is detectable after stage 6 (Figure 1C). Again, these *awd* mutant cells have smaller nuclei (insets in Figure 1C). Consistent with increased proliferation in *awd* mutant follicle cells, prolonged expression of the mitotic marker cyclin B was also detected in these mosaic ovaries (GFP-negative cells are mutants in Figure 2A). Note that while cyclin B is absent in *awd*^*+*^ cells, in *awd* mutant cells, cyclin B is not uniformly expressed at high levels. This is likely because the cell cycle is not synchronized in all follicle cells. In addition, the known Notch down-regulation target *cut*[40] is over-expressed in Awd-negative cells (Figure 2B). Compromised Notch signaling also results in expression of immature cell-fate markers in follicle cells beyond stage 6. In wild-type egg chambers Fasciclin III (FasIII) is expressed in all follicle cells up to stage 3 of oogenesis and then becomes restricted to the polar follicle cells (PC in Figure 2C) at the anterior and posterior poles of the follicular epithelium. Reduction of Notch activity arrests follicle cells in an undifferentiated state and up-regulates FasIII expression [42]. Follicle cell clones mutant for *awd* show strong expression of FasIII after stage 6, indicating that they are defective in terminal differentiation (*awd* mutant cells lacking GFP expression in Figure 2C). Down-regulation of *cut* in wild-type follicle cells is mediated by Hindsight (Hnt), an up-regulation target of Notch [40,47]. To examine loss of Notch target gene expression, we used the MARCM method of clonal analysis, which results in GFP-expression in mutant cells, so as to ensure that lack of gene expression is not the result of cell death (Figure 2D,E). In contrast to wild-type follicle cells, the MARCM clone of *awd* mutant cells (GFP-positive) does not express Hnt after stage 6 (Figure 2D). To further confirm that Notch signaling is attenuated in *awd* mutant follicle cells, the expression of *GbeSu(H)*_*m8*_*-lacZ* transcriptional reporter for Notch activity [48] was examined. In MARCM *awd* clones, β-galactosidase staining is absent or strongly reduced (GFP-positive cells in Figure 2E).

**Figure 3:**
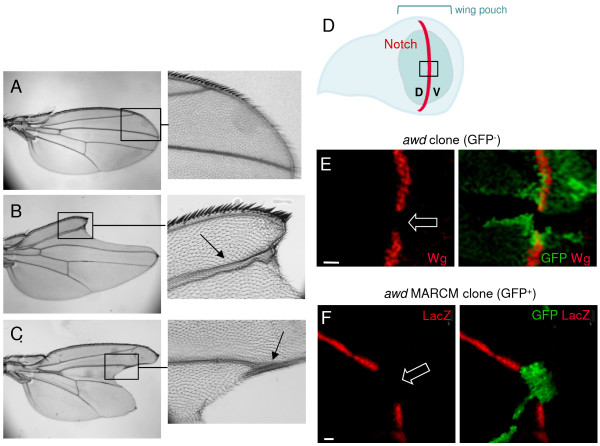
**Notch signaling defect in adult wings and larval wing discs.** Compared to *yw* flies, representing wild-type **(A)**, wings from flies of the genotype *en2.4-Gal4*^*e22c*^*, UAS-flp/+; FRT*^*82B*^*/FRT*^*82B*^*, awd*^*j2A4 *^**(B-C)** show typical *Notch* phenotypes: enlarged wing veins (arrows) and loss of wing margins (‘notched’ wing blades). **(D)** Drawing of a third instar wing disc in apical view showing the dorsal-ventral (D, V) compartment border (red line) specified by the Notch activity. The wing disc pouch is the central fold of the disc (green) and will generate the wing blade. The black box approximately indicates the areas shown in E and F. **(E)** The discs were dissected from *hs-flp; +/+; FRT*^*82B*^*, Ubi-GFP/FRT*^*82B*^*, awd*^*j2A4*^ third instar larvae. *wingless* (*wg*) is a downstream activation target of *notch*. Wg protein expression is lost in the *awd* clone (loss of GFP; empty block arrow) overlapping the midline (dorsal-ventral boundary, where Notch specifies *wg* expression). **(F)** The discs were dissected from *hs-flp/GbeSu(H)*_*m8*_*-lacZ; act-Gal4, UAS-GFP/+; FRT*^*82B*^*, act-Gal80/FRT*^*82B*^*, awd*^*j2A4*^ third instar larvae. *GbeSu(H)*_*m8*_*-lacZ* expression (red) is also lost in *awd* MARCM clones (expressing GFP; empty block arrow). Bars are 10 μm.

The Notch signaling defect in *awd* mutant cells suggested a potential mechanism for the original defining phenotype of *awd* - abnormal wing discs, because during development Notch specifies the dorsal-ventral margin of the wing discs (which becomes the wing peripheral margin in the adult) and the vein-intervein boundary, and is important for disc cell proliferation. Loss of *Notch* function causes wing margin defects and widening of wing veins [26]. As shown in Figure 3, 72% (18/25) of adult mosaic flies show typical *Notch* phenotypes in wings with ‘notched’ wing margins and wing vein thickening (Figure 3A-C). In wild type wing discs, activation of the Notch pathway at the dorsal-ventral boundary (Figure 3D) leads to the expression of target gene products, such as the signaling molecule Wingless (Wg) [49]. Loss of *awd* function abolished the Wg staining in third instar wing disc clones at the dorsal-ventral boundary (GFP-negative cells in Figure 3E). To further verify the Notch signaling defect, we examined *GbeSu(H)*_*m8*_*-lacZ* reporter expression using a different mosaic fly generated by the MARCM system. Similar to our results in follicular epithelium, β-galactosidase expression in *awd* mutant clones (GFP-positive cells) in the dorsal-ventral boundary is lost (Figure 3F).

### *awd* function is required for signaling after the S2 cleavage of Notch

In the egg chamber, Notch functions in the follicle cells while the ligand Delta is expressed in the abutting germline cells [42]. Since the *awd*^*j2A4*^ clones were induced specifically in follicle cells, the defective Notch signaling in mutant follicle cells is not likely to be the result of a defect in Delta expression or endocytosis in the abutting germline cells. Also importantly, in *Delta* mutant NICD antibody-detected Notch accumulates on the follicle cell surface, which is consistent with the notion that ligand binding precedes intracellular trafficking and proteolytic processing of Notch [42].

**Figure 4:**
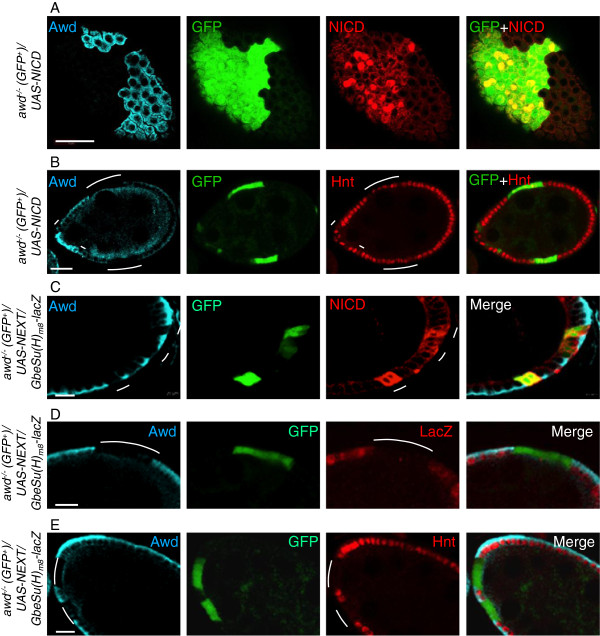
**Notch signaling defect in *awd *mutant cells is rescued by exogenous NICD.** Stage 7–8 egg chambers were dissected from females of the genotype *hs-flp/GbeSu(H)*_*m8*_*-lacZ; act-Gal4, UAS-GFP/UAS-NICD; FRT*^*82B*^*, act-Gal80/FRT*^*82B*^*, awd*^*j2A4 *^**(A-B)** or *hs-flp/GbeSu(H)*_*m8*_*-lacZ; act-Gal4, UAS-GFP/UAS-NEXT; FRT*^*82B*^*, act-Gal80/FRT*^*82B*^*, awd*^*j2A4 *^**(C-E)**. **(A, C)** As controls, NICD and NEXT expression is verified. **(B)** A stage 7 egg chamber with MARCM clones of *awd* (Awd-negative and GFP^+^, marked by lines) simultaneously expressing NICD. The expression of endogenous Hnt (red) is restored in a majority of the mutant cells. The Awd staining is in cyan. Note that the diffused Awd staining in regions abutting the apical side of the follicle cells is within the germline cells, occasionally observed in abnormal egg chambers. **(D-E)** Exogenously expressed NEXT cannot rescue the *awd* mutant phenotype. *awd* MARCM mutant clones lacking Awd staining (cyan) marked by the GFP expression and indicated by lines show loss of *GbeSu(H)*_*m8*_*-lacZ* reporter gene expression (red in **D**) as well as loss of Hindsight (Hnt) expression (red in **E**). Bars in **(A-B)** are 20 μm. Bars in **(C-E)** are 10 μm. NEXT, Notch external truncation; NICD, Notch intracellular domain.

To define the step where Notch signaling is stalled in *awd* mutant follicle cells we over-expressed NICD or NEXT in *awd* mutant follicle cells by using the MARCM system. NICD is the cytoplasmic domain of Notch that functions as a cytoplasmic, γ-secretase-independent constitutively active Notch, while NEXT is the truncation generated after the S2 cleavage devoid of the ligand-binding domain, S2 cleavage site, and the negative-regulatory region (NRR) [50,51]. NEXT is a membrane-bound, γ-secretase-dependent, constitutively active form of Notch that can function without ligand but still requires intracellular proteolytic processing and trafficking [52]. To assess rescue of Notch signaling we analyzed the Hnt expression (no expression in *awd* mutant). In stage 7–8 *awd* clones over-expressing NICD (from the *UAS-NICD* transgene [53]) (GFP-positive cells in Figure 4A), 60.5% of mutant cells express Hnt (199 out of 329 cells) (GFP-positive cells in Figure 4B), representing a significant rescue of the lack of Hnt expression phenotype. This is also consistent with the observation that the over-expressed NICD is localized in the nuclei in a significant number of *awd* mutant follicle cells (Figure 4A). Furthermore follicle cells flp-out clones expressing the same NICD transgene also show enhanced Hnt expression at stage 7–8 [see Additional file 1: Figure S1A], as well as enhanced the size of nuclei at stage 10B (not shown) [54]. In contrast, expression of the *UAS-NEXT* transgene [55] in the *awd* clone (GFP-positive cells in Figure 4C) did not rescue Notch signaling as assessed by loss of *GbeSu(H)*_*m8*_*-lacZ* expression (GFP-positive cells in Figure 4D) and loss of Hnt expression (GFP-positive cells in Figure 4E). The same transgene is able to upregulate the Hnt expression in flp-out clones of follicle cells [see Additional file 1: Figure S1B]. Note that the over-expressed NEXT accumulates in the intracellular vesicles (Figure 4C), consistent with the notion that internalization of surface Notch can occur in *awd* mutant cells but the subsequent vesicle trafficking is defective.

It has recently been shown that transmission of Notch signal requires proper intracellular trafficking, at least in *Drosophila* follicle cells and imaginal discs [32-34,55]. Therefore, our observed Notch processing and signaling defects may result from either defective proteolytic cleavage of Notch to release intracellular domain by γ-secretase or defective endocytic transport of Notch. We favor the latter mechanism since Awd has been shown to promote endocytosis of surface receptors in multiple tissues [21-25]. In addition, neither the expression level nor the punctate expression pattern of Presenilin [56-58], the catalytic component of the γ-secretase complex, are altered in *awd* mutant follicle cells [see Additional file 2: Figure S2]. To test the notion that the Notch signaling deficiency in *awd* mutant cells is the result of defective endocytosis, we next examined the localization of Notch receptor in *awd* follicle cell clones.

### Notch accumulates in endocytic vesicles in *awd* mutant cells

**Figure 5:**
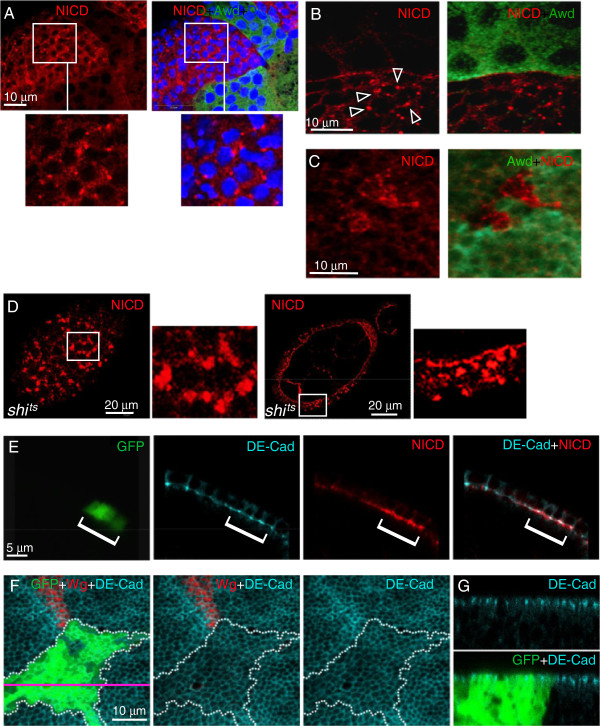
**Defective intracellular distribution of Notch in *awd *mutant cells. (A-B)** Stage 8 egg chambers were dissected from *hs-flp; +/+; Ubi-GFP, FRT*^*82B*^*/FRT*^*82B*^*, awd*^*j2A4*^ females, and stained for NICD (red), Awd (green) and DNA (blue). Notch over-accumulates in vesicles near the cell periphery (insets in **(A)** and arrowheads in **(B)**). **(C)** The wing disc was dissected from *hs-flp; +/+; Ubi-GFP, FRT*^*82B*^*/FRT*^*82B*^*, awd*^*j2A4*^ third instar larva and stained for NICD (red). *awd* clones were identified by lack of Awd staining (pseudo-colored in green). Notch in *awd* mutant clones accumulates in large vesicles. **(D)** Surface and cross-section views of *shi*^*ts*^ stage 7 egg chambers from females incubated at 29°C and stained for NICD. Very large aggregates are seen on the surface and throughout the cells. **(E)** A stage 7 egg chamber from a *hs-flp/GbeSu(H)*_*m8*_*-lacZ; act-Gal4, UAS-GFP/+; FRT*^*82B*^*, act-Gal80/FRT*^*82B*^*, awd*^*j2A4*^ female was stained for DE-cadherin (cyan) and NICD (red). Notch accumulates in *awd* mutant cells (GFP-positive) that show normal DE-cadherin distribution. **(F-G)** Third instar wing imaginal disc dissected from a *hs-flp/+; act-Gal4, UAS-GFP/+; FRT*^*82B*^*, act-Gal80/FRT*^*82B*^*, awd*^*j2A4*^ larva and stained for DE-cadherin (cyan) and Wg (red) in which the *awd* mutant clone is marked by GFP expression and outlined in F by the dotted area. **(F)** The confocal section of the apical region of disc cells (x-y) shows that *awd* loss of function does not affect the distribution of DE-cadherin. **(G)** The cross section through the disc epithelium (x-z) with apical side up also shows an unaffected apical/basal polarity distribution of DE-cadherin in *awd* mutant cells. The pink line indicates the position of the x-z section. Awd, Abnormal wing discs; NICD, Notch intracellular domain.

While in *awd*^*+*^ cells Notch is present in low abundance in small punctates, Notch accumulates in large vesicle-like aggregates near the apical surface in *awd* mutant clones in follicular epithelium (Figure 5A,B) and in wing discs (Figure 5C). Such Notch accumulation phenotype in *awd* mutant resembles that of mutants in *avalanche* (*avl;* which encodes Syntaxin) and *rab5*[34,55,59], two gene functions required for maturation of early endosomes [59], but is different from the phenotype in *dynamin* mutant (*shi*^*ts*^), in which Notch accumulates on the cell surface and in very large aggregates on apical and basal sides of the follicle cells (Figure 5D) as noted previously [60]. This pattern is likely because of the failure to deliver Notch to apical membrane via Dynamin-mediated transcytosis [61] as well as to internalize Notch for signaling [55]. Since *awd* mutant cells do not show these very large aggregates throughout the cells, it is unlikely that *awd* function completely overlaps with that of *dynamin*. Notch localization can also be influenced by the integrity of the adherens junction [61]. Since we have shown previously that the *awd* mutant can affect the membrane localization of E-cadherin and β-catenin [25], we also determined that Notch localization defect not only occurred in *awd* mutant pile-up epithelial cells [see Additional file 3: Figure S3] but also occurred in *awd* mutant follicle cells that show normal epithelial polarity, indicated by normal E-cadherin localization (Figure 5E). *awd* mutant clones exhibiting normal epithelial integrity are most often observed in clones of small size (<10 cells; unpublished observation). We showed that small *awd* mutant clones indeed lacked Hnt expression [see Additional file 4: Figure S4]. We also showed that the epithelial polarity of *awd* mutant cells in wing disc is unaffected as shown by normal E-cadherin localization (Figure 5F,G in which GFP^+^ cells are *awd* mutants). Since Notch processing in the follicle cells has been shown to occur during transition from mature early endosomes to late endosomes [55,62], we suspected that the endocytosis defect in *awd* mutant cells might be in the step prior to the formation of late endosomes.

**Figure 6:**
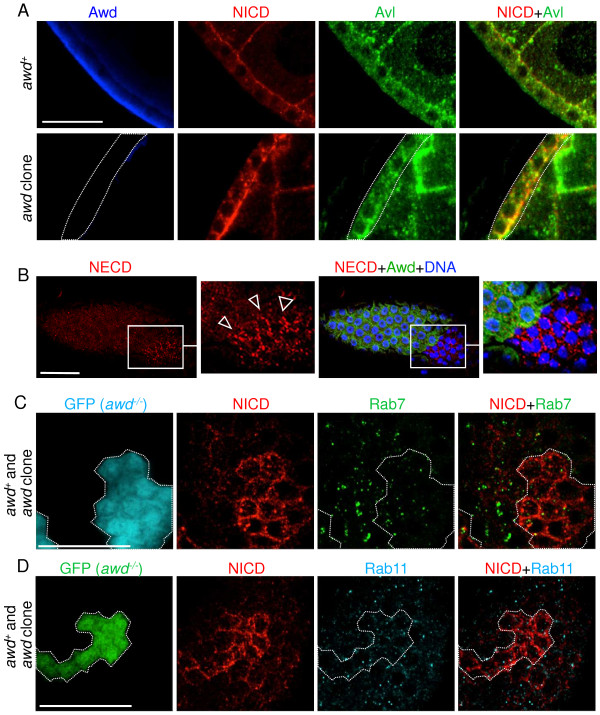
**Notch accumulates in early endosomal compartments in *awd *mutant cells. (A)** Stage 8 egg chambers were dissected from *yw* (wild-type; upper panel) or *yw; en2.4-Gal4*^*e22c*^*, UAS-flp/+; FRT*^*82B*^*/FRT*^*82B*^*, awd*^*j2A4*^ (*awd* clone, lower panel) females. In wild-type, Notch shows low level punctates that are partially co-localized with Avl. In the *awd* clone, most, if not all, large Notch-positive vesicles are also Avl-positive. Dashed line marks the *awd* clone. **(B)** Surface view of stage 7 egg chamber dissected from a *yw; en2.4-Gal4*^*e22c*^*, UAS-flp/+; FRT*^*82B*^*/FRT*^*82B*^*, awd*^*j2A4*^ female and stained for Notch extracellular domain peptide (NECD), Awd and DNA. There is accumulation of NECD on the surface of *awd* mutant clones (empty arrowheads). **(C-D)** Stage 8 egg chambers were dissected from *hs-flp/GbeSu(H)*_*m8*_*-lacZ; act-Gal4, UAS-GFP/+; FRT*^*82B*^*, act-Gal80/FRT*^*82B*^*, awd*^*j2A4*^ females. *awd* mutant clones were identified as GFP-expressing cells. In *awd* clones, over-accumulated Notch does not co-localize with Rab7 **(C)** or Rab11 **(D)**. Bars are 20 μm. Avl, avalanche; Awd, abnormal wing disc.

To verify this notion, we first examined Notch localization in the endocytic pathway in *awd* mutant cells. In *awd*^*+*^ cells, NICD is in small punctates with partial co-localization with Avl, a component of the early endosome (Figure 6A, upper panels), consistent with previous observations [34,55]. In *awd* mutant cells, the level of Notch-Avl colocalization increased by 2 fold (Figure 6A, bottom panels; statistical analysis reported in Additional file 5: Figure S5A,A’).

In order to determine whether these Avl-positive, Notch-containing vesicles are immature early endosomes that cannot form multivesicular bodies (MVBs), we examined the *awd* mutant vesicles in relation to hepatocyte growth factor-regulated tyrosine kinase substrate (Hrs), which is involved in the maturation of early endosomes by promoting ubiquitinated cargo sorting [63]. It marks the mature early endosomes and MVBs. We observed similar, low-level co-localization of Notch and Hrs in both *awd*^*+*^ and *awd* mutant cells [see Additional file 5: Figure S5B,B’ for statistical analysis]. Lack of significant Notch-Hrs co-localization even in *awd*^*+*^ cells is consistent with the finding that normal Notch signaling is not affected in *hrs* mutants [55]. Some co-localization of Hrs and Notch in *awd* mutant cells is also consistent with the observation that a minor Rab5-independent route exists for Notch sorting [55]. On the other hand, this Notch accumulation pattern is very different from that of the *phyllopod* mutation which blocks Notch entry into late endosomes but not entry into mature early endosomes, resulting in increased Notch signaling and significant co-localization of NICD and Hrs [64]. This suggests that early endosome maturation is defective in *awd* mutant cells.

Since *awd* can also act on the internalization of surface receptor [21], we examined whether constitutive internalization of full-length Notch is affected in *awd* mutant cells. This was detected by using an antibody against the NECD. As shown in Figure 6B, NECD antibody indeed detected increased accumulation of full-length Notch in *awd* mutant cells. Therefore, Awd can act on both internalization of surface Notch and intracellular trafficking of signaling Notch.

### Notch does not traffic to late endosomes in *awd* mutant cells

It has been shown that Notch signaling can also be enhanced by blocking MVB formation with mutations in the *endosomal sorting complex required for transport* (*ESCRT*) genes *tsg101*, *vps25* and *vps20*, or by promoting early endosome maturation with over-expression of constitutively active Rab5 [55]. Since the *awd* mutant is defective in Notch signaling, it is unlikely that the Notch-containing vesicles in *awd* mutant cells have passed into late endosomes. This notion is supported by the lack of significant co-localization of Notch-containing vesicles in MARCM *awd* mutant clones with Rab7, the late-endosomal marker (GFP-positive cells in Figure 6C; statistical analysis reported in Additional file 6: Figure S6). As well, transition from early endosomes to late endosomes is accompanied by acidification of the luminal contents, which can be detected by Lysotracker staining. Consistent with the notion that Notch-containing vesicles in *awd* mutant cells cannot enter MVB and late endosomes, we observed no difference in Lysotracker-positive vesicles in *awd*^*+*^ and *awd* mutant cells [see Additional file 7: Figure S7]. In addition, the Notch-containing vesicles in MARCM *awd* mutant clones are not Rab11-positive recycling endosomes, either (GFP-positive cells in Figure 6D; see Additional file 8: Figure S8 for statistical analysis).

**Figure 7:**
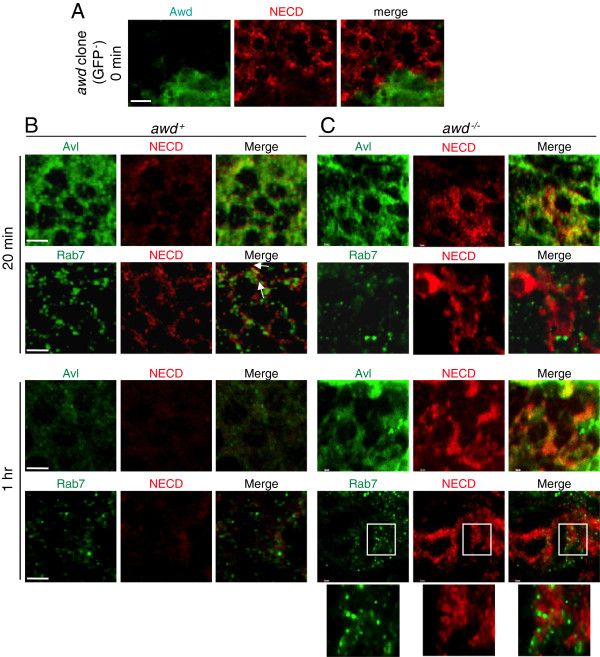
**Endocytic defects in *awd *mutant cells.** Notch trafficking assay was performed on wing discs from **(A, C)***yw; en2.4-Gal4*^*e22c*^*, UAS-flp/+; Ubi-GFP, FRT*^*82B*^*/FRT*^*82B*^*, awd*^*j2A4*^ and **(B)***yw* third-instar larvae. **(A)** In a disc containing *awd* mutant clone (GFP-negative cells) without synchronized induction of endocytosis (steady state time 0), Notch is seen over-accumulating on or near the cell surface. **(B)** In wild-type (*yw*) discs, at 20 minutes after initiation of endocytosis, Notch is expressed at a low level in punctates that are mostly localized in Avl-positive vesicles with some localization in Rab7-positive vesicles (arrows). At one hour after initiation of endocytosis, Notch is barely detectable. **(C)** In *awd* mutant clones, at 20 minutes after initiation of endocytosis, Notch over-accumulates exclusively in Avl-positive vesicles but not in Rab7-positive vesicles. This pattern persists at one hour after initiation of endocytosis. The accumulated Notch at this time does not overlap with Rab7-positive vesicles (insets). Bars are 5 μm.

We next sought to follow the time course of Notch localization in live cells. Wing discs are an ideal and standardized system for this purpose since they can be cultured *ex vivo* for a prolonged period of time. Note that in this established *ex vivo* system, internalization of Notch is detected by binding to NECD antibody, without binding to spatially-expressed ligands. Therefore, the system strictly measures the kinetics of vesicular transport, not the endogenous signaling process. We first established that at the steady state (time 0), Notch accumulated on the *awd*^*−/−*^ cell surface (Figure 7A). In wild-type cells, internalized Notch follows a typical time course: at 20 minutes after initiation of endocytosis, Notch is mostly in Avl-positive early endosomes while some has passed into Rab7-positive late endosomes (Figure 7B). At one hour after endocytosis, the Notch signal is barely detectable, consistent with the degradation time course. Also, in wild-type cells, Avl staining is much more pronounced at 20 minutes than at one hour. This is likely because in this label-and-chase experiment, a large number of Avl-positive vesicles were formed synchronously after initiation of endocytosis. Concentrated Avl was then lost (therefore detected at a lesser extent by immunofluorescence) after early endosomes matured and were incorporated into late endosomes.

In *awd* mutant, on the other hand, accumulated Notch is mostly on cell surface or in Avl-positive early endosomes at 20 minutes and remains in these early endosomes even one hour after internalization (Figure 7C). The Notch signal shows no localization to the late endosomes (Figure 7C). Note that some of the Rab7-positive vesicles shown in Figure 7C are very close to or surrounded by the Notch signal but are not overlapping (Figure 7C insets).

### *awd* is required for Rab5 function

**Figure 8:**
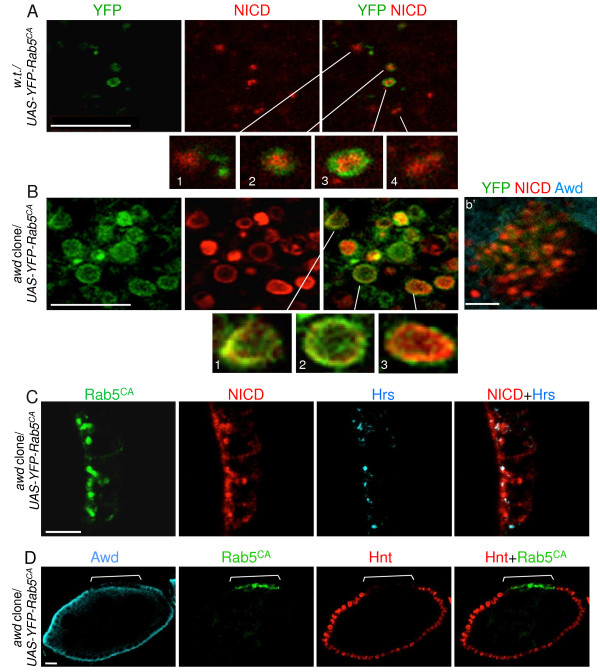
**Awd is required for Rab5 function.** YFP-tagged constitutively active Rab5 mutant Q88L (Rab5^CA^) were expressed in **(A)** wild-type or **(B)***awd* mutant clones, using the genetic combinations *en2.4-Gal4*^*e22c*^*, UAS-flp/UAS-YFP-RAB5*^*Q88L*^*; +/FRT*^*82B*^ or *en2.4-Gal4*^*e22c*^*, UAS-flp/UAS-YFP-RAB5*^*Q88L*^*; awd*^*j2A4*^*, FRT*^*82B*^*/FRT*^*82B*^*,* respectively*.* Egg chambers were processed for staining for NICD (red) and YFP (green) as indicated. *awd* mutants were verified by lack of Awd staining (cyan in **b**’). **(A)** YFP-Rab5^CA^ expressed in wild-type follicle cells. In Rab5^CA^-expressing wild-type follicle cells, NICD is reduced and is present in either Rab5-positive (insets 2 and 3) or Rab5-negative (likely late endosomes; insets 1 and 4). Note that NICD is in the lumen of these vesicles. **(B)** YFP-Rab5^CA^ expressed in *awd* mutant follicle cells. In Rab5^CA^-expressing *awd* mutant cells, abundant NICD is present in enlarged vesicles that are mostly Rab5-positive. NICD is enriched on the surface of these vesicles (insets 1–3). **(C)** A stage 8 egg chamber from *hs-flp/GbeSu(H)*_*m8*_*-lacZ; UAS-YFP-Rab5*^*CA*^*/+; tub-Gal4, FRT*^*82B*^*, tub-Gal80/FRT*^*82B*^*, awd*^*j2A4*^ was stained for Hrs (cyan), YFP (green) and NICD (red). There is only partial co-localization of accumulated Notch with Hrs. **(D)** A stage 8 egg chamber from *hs-flp/GbeSu(H)*_*m8*_*-lacZ; UAS-YFP-Rab5*^*CA*^*/+; tub-Gal4, FRT*^*82B*^*, tub-Gal80/FRT*^*82B*^*, awd*^*j2A4*^ was stained for Awd (cyan), YFP (green) and Hnt (red). Expression of Rab5^CA^ in Awd-negative cells (bracket) cannot rescue the loss of Hnt expression. Note that the Awd positive signal apical to the *awd* mutant clone is the expression sometimes detectable in the germ cell abutting the *awd* mutant clones. Bars are 10 μm. Hrs, hepatocyte growth factor-regulated tyrosine kinase substrate; NICD, Notch intracellular domain; YFP, yellow fluorescent protein.

To further test the role of *awd* in early endosome maturation, we next tested how expression of constitutively active Rab5 (Rab5^CA^) might affect Notch localization in *awd* mutant. As mentioned above, Rab5^CA^ has been shown to increase Notch signaling [55], presumably because the endocytic process is pushed through early endosomes. In *awd*^*+*^ cells, NICD is found in both Rab5^CA^-positive (Figure 8A, insets 2 and 3) and -negative (Figure 8A, insets 1 and 4) vesicles, and, importantly, the detectable NICD is almost exclusively in the lumen of these vesicles. The likely interpretation is that Rab5^CA^ pushes endocytosis through early endosomal stages and Notch is processed. Processed endogenous NICD becomes diffused in the cytosol and nuclei, and undetectable by immunohistochemistry (IHC) in our assay system. Remaining, predominantly luminal, NICD is an unprocessed subpopulation that is internalized in the MVBs or late endosomes destined for degradation [31,62]. Strikingly, in *awd* mutant clones, NICD is found exclusively in the Rab5^CA^-positive vesicles (Figure 8B). Most importantly, in *awd* mutant cells, much of the NICD signal is mostly present on the surface of these enlarged vesicles (Figure 8B insets 1–3). The result indicates that although cell surface-bound Notch can be internalized in *awd* mutant cells in the presence of Rab5^CA^, it is not processed and cannot enter late endosomes. In addition, in *awd* mutant follicle cells 87.1% of Notch vesicles co-localize with Rab5^CA^ and 31.45% co-localize with Hrs (n = 124) (Figure 8C; see Additional file 9: Figure S9 for co-localization analysis). Co-localization of NICD and Hrs in *awd* mutant cells increases by the over-expression of Rab5^CA^ [see Additional file 9: Figure S9B compared to Additional file 5: Figure S5B’]. This suggests that Rab5^CA^ partially stimulates vesicles to progress through the endocytic pathway but *awd* function is necessary for Rab5-mediated early endosome maturation. This notion is supported by the increased number of Rab5^CA^-positive vesicles in *awd* mutant clones (Figure 8B), indicating a block in vesicle trafficking downstream of Rab5 function. This interpretation is confirmed since Rab5^CA^ cannot rescue Notch signaling in *awd* mutant cells (Figure 8D).

Taken together, these results suggest that during Notch signaling *awd* function is downstream of or is required for Rab5 function in promoting maturation of early endosomes.

## Discussion

In this report we demonstrate a role of *awd* in regulating Notch signaling via its endocytic function including surface internalization and vesicle trafficking. This conclusion is based on our results that show: (1) multiple Notch target genes are mis-expressed in follicle cells and wing discs; (2) Notch accumulates in enlarged early endosomes; and (3) *awd* function is required for the Rab5 activity in early endosome maturation. Our results also indicate that during vesicles trafficking, the Awd action is downstream of the S2 cleavage, since over-expressed of NEXT accumulated intracellularly and could not rescue the *awd* defect. The same NEXT over-expression strategy could rescue the *shi/dynamin* defect [57,65], strongly supporting the notion that the Awd action on Notch signaling is post-membrane invagination. Since over-expression of NICD could rescue the *awd* defect, the Awd action is likely upstream or in parallel to the S3 cleavage event (γ-secretase activity). Although a role of *awd* in promoting the activity of γ-secretase cannot be completely ruled out, we considered this possibility unlikely. First, *awd* is a known endocytic factor demonstrated in multiple tissues including neurons, trachea, and follicle cells [22-25]. Second, neither the expression level nor the expression pattern of Presenilin, the catalytic subunit of γ-secretase, is altered in *awd* mutant cells. Third, if the defect is in γ-secretase function, it would be expected that Notch should accumulate in Hrs-positive MVBs [60]. On the contrary, we did not observe such ectopic accumulation of Notch in Hrs-positive vesicles. Therefore, our results, in aggregate, suggest that the main action of Awd on Notch signaling is via its endocytic activity promoting the transition from early endosomes to late endosomes. However, potential defects downstream of γ-secretase cleavage, such as trafficking to nucleus, in *awd* mutant cannot be formally ruled out.

One curious exception for the *awd* function in relation to Notch signaling is found in the border cells. As we have recently reported [24], during the migration of these cells, Awd expression is down-regulated. Re-expression of Awd can lead to reduction of surface receptors, such as PVR that is critical for directional movement, resulting in defective migration. Interestingly, Notch signaling is also important for border cell migration [66]. It, therefore, appears that Notch signaling in these specialized cells does not require Awd activity or is insensitive to Awd protein levels. To test this, we compared Notch expression in border cells with or without Awd re-expression. In wild-type border cells (no Awd), Notch is located on the cell surface as well as in the cell body, consistent with active signaling (data not shown). Forced re-expression of Awd in the border cells does not alter this pattern. This may be because Notch is already actively internalized; increasing the Awd level cannot further enhance such activity. Indeed, endocytosis is intrinsically highly active in border cells [24,67]. Alternatively, the differential dependence of Notch on Awd activity may be a function of how Notch is activated, not how Awd functions differently in different cell types. For example, Dobens *et al*. [68] have shown that the Notch ligand Delta may be co-expressed with Notch in the same border cells. Recent reports have hinted that the requirement of endocytosis for Notch signaling may depend on the ligand-receptor relationship (for example, ligand-dependent or -independent, trans- or cis-activation, and so on) [62]. We, therefore, consider that the apparent Awd-independent Notch signaling in border cells has more to do with the intrinsic Notch signaling mechanism in these cells, and less to do with the function of Awd.

Our results indicate that the Notch signaling defect in *awd* mutant cells is the failure to deliver Notch past the Rab5-dependent early endosomal stage. On the other hand, the ESCRT complex mutants, which are defective in late endosome formation, promote Notch signaling [34,55]. Taken together, it appears that Notch activation occurs in the intermediate stage between early endosome formation and late endosome entry. Transition from early endosomes to late endosomes is accompanied by cargo sorting, intravesicular invagination and acidification of the luminal contents. Curiously, the matured early endosome and MVB marker *hrs* mutant has no effect on Notch signaling [55], which indicates that endosomal cargo sorting *per se* is not required for Notch signaling. We have also shown that *awd* mutant cells do not exhibit altered levels of Lysotracker staining and that endosomal Notch remains on the surface of enlarged endosomes in *awd* mutants. The exact nature of this transition state that favors Notch processing, therefore, requires further analysis. The endocytic function of *awd* has traditionally been described as a ‘GTP supplier’ for Dynamin, based on genetic interaction data and logical extrapolation because of the GTP producing activity of Awd [22]. In this report, we demonstrate that, in relation to Notch signaling, *awd* functions downstream of, but not directly on, *dynamin*. It is instead critical for Rab5 activity. This is supported by the following evidence: 1) Notch in *awd* mutant accumulates in Avl-containing vesicles. Therefore, the *awd* defect is post Dynamin-mediated cleavage of membrane invagination. 2) Rab5^CA^ can push Notch into enlarged early endosomes but failed to rescue the *awd* phenotype, thereby strengthening the notion that *awd* defect is post Shi/Dynamin function. 3) The Notch accumulation pattern in *shi* mutant is different from that in *awd* mutant. 4) Over-expression of NEXT could not rescue *awd* defect. The same NEXT over-expression strategy could rescue the *shi* defect, strongly supporting the notion that the Awd action concerning Notch signaling is post-membrane invagination [57,65]. It should be noted that we did observe surface accumulation of NECD antibody-detected Notch molecules, likely representing the full-length Notch not engaged in ligand binding and signaling. This indicates that Awd can affect constitutive internalization of full-length Notch.

The requirement of endocytosis in the signal-receiving cells for Notch activation has been amply demonstrated [69]. It has been shown that Notch signaling in follicle cells after stage 6 requires Delta [42]. Since in this report we show that Notch signaling cannot occur in the follicle cell without *awd* function, we conclude that, at least in follicle cells, endocytosis is a requisite process for ligand-dependent Notch signaling.

The involvement of endocytosis in Notch signaling is significant since many of the endocytic components shown to regulate Notch signaling have also been implicated in carcinogenesis. For example, V-ATPase is required for Notch signaling while mutations in ESCRT components, such as Tsg101, result in increased Notch signaling. V-ATPase has generally been considered an oncogene [70] because it is associated with acidification of tumor cells. ESCRT components, on the other hand, have been shown to suppress tumor formation because they down-regulate surface growth factor receptor signaling [71]. As such, attempts to design therapeutics based on these prevalent functions should take into account the effects on Notch signaling, since the relationship between Notch signaling and carcinogenesis is context-dependent [35,72,73].

## Conclusions

Awd belongs to the Nm23 family of protein that is evolutionarily conserved from *Drosophila* to mammals. Our *in vivo* analyses demonstrate that loss of *awd* gene function blocks Notch signaling by altering the receptor processing after the S2 cleavage and causes Notch accumulation in early endosomes. Furthermore, we obtained evidence indicating that Awd is required for Rab5 function in early endosome formation.

*Nm23* has been an enigmatic gene function. It is a housekeeping gene involved in nucleotide synthesis and energy metabolism, and yet exhibiting specific developmental functions [6,24]. It was the first metastasis suppressor gene identified [4,74], yet exhibits oncogenic functions in some cancer cohorts [9,10]. We have previously shown that either loss-of-function or over-expression of *awd* can affect different aspects of epithelial morphogenesis. That is, loss-of-function *awd* results in over-accumulation of adherens junction components and piling up of the epithelium, while over-expression of *awd* results in reduced adherens junctions and disintegration of epithelial structure [25]. These findings provided some explanation of the biphasic function of Nm23 in tumorigenesis. In light of the studies presented here, an additional level of complexity should be considered since Notch signaling can exert different cellular functions in different tissues and at different times during pathophysiological alterations of the same tissues [35].

## Methods

### *Drosophila* strains and genetics

Stocks were raised on standard cornmeal/yeast/agar medium at 25°C. The stock carrying the protein-null *awd* allele, *awd*^*j2A4*^, has been described [22-25]. The *awd*^*j2A4*^ allele combined with the *FRT* chromosome *FRT*^*82B*^ has been described [24,25]. Cell clones mutant for *awd*^*j2A4*^ were generated through mitotic recombination using the FLP/FRT system [44], either with the *hs-flp* recombinase transgene or using the directed mosaic technique with the *UAS-flp* transgene under control of the ubiquitous somatic cell driver *en2.4-Gal4*^*e22c*^[45]. To obtain over-expression of specific transgenes in *awd*^*j2A4*^ mutant follicle cells we used either the directed mosaics or the MARCM [46] techniques. The transgenic line carrying the constitutively active (CA) variant of the YFP-Rab5 fusion genes was obtained from the Bloomington Stock Center (Bloomington, IN, USA) [75]. The *UAS-NICD* and the *GbeSu(H)*_*m8*_*-lacZ* lines were a kind gift from S. Bray of University of Cambridge (Cambridge, UK). The *UAS-NEXT* line was a kind gift from M. Fortini of Thomas Jefferson University (Philadelphia, PA, USA). The genotypes of flies and larvae used for the analyses are described in Additional file 10; Supplementary experimental procedures.

### Immunohistochemistry

Ovaries were dissected, fixed and stained as previously described [76] with the exception of ovaries from *shi*^*2*^*/shi*^*2*^ (*shi*^*ts*^) females that were fixed at 29°C. Whole late third instar larvae were dissected into room temperature PBS (pH 7.5), and fixed for 20 minutes in 4% formaldehyde. After three washes in PBS, larval tissues were permeabilized for one hour in PBT (0.3% Triton X-100 in PBS) and then were blocked in 2% BSA in PBT for 10 minutes at room temperature. Overnight incubation at 4°C with primary antibodies in 2% BSA in PBT was followed by three washes in PBT and 10 minutes incubation in 2% BSA in PBT. Larval samples were then incubated with fluorescence-tagged secondary antibodies for two hours at room temperature and after extensive washes in PBT the wing discs were dissected. Primary antibodies used are: chicken anti-Avl (1:500) [59], mouse monoclonal anti-NICD (1:1000; C17.9c6, Developmental Studies Hybridoma Bank (DSHB, Iowa City, Iowa, USA)), mouse monoclonal anti-NECD (1:50; C458.2H, DSHB), mouse monoclonal anti-Cut (1:15; 2B10, DSHB), mouse monoclonal anti-Hnt (1:30; 1G9, DSHB), mouse monoclonal anti-Cyclin B (1:100; F2F4, DSHB), rat monoclonal anti-DE-cadherin (1:100; DCAD2, DSHB) and mouse monoclonal anti-β-gal (1:25; 40-1A, DSHB); and protein A-purified rabbit anti-Awd (1:2000) [23], rabbit anti-phosphohistone H3 (1:200; 06–570, Upstate Biotechnology, Lake Placid, NY, USA), rabbit anti-C-Psn (1:200) [58], rabbit anti-Rab7 (1:2000) and rabbit anti-Rab11 (1:8000) [77]. Secondary antibodies used are: Cy3- (1:100; Jackson Lab, West Grove, PA, USA), DyLight 649- (1:200; Jackson Lab), or FITC- (1:250; Invitrogen, Molecular Probes, Eugene, OR, USA) conjugated anti-mouse immunoglobulin G (IgG); and Cy3- (1:1000; Sigma, Saint Louis, Missouri, USA), DyLight 649- (1:500; Jackson Lab), or BODIPY- (1:2000; Molecular Probes) conjugated anti-rabbit IgG.

DNA staining was carried out by incubating egg chambers and wing discs for 10 minutes with 4',6-diamidino-2-phenylindole (DAPI; Sigma) at 0.5 μg/ml in PBS followed by several washes with PBS. To-Pro-3 (Molecular Probes) nuclear staining was also carried out after immunodetection by incubating the egg chambers for two hours with To-Pro-3 at 1 μM in PBS on a rotating wheel followed by several washes with PBT. Stained egg chambers or wing discs were mounted in Fluoromount-G (Electron Microscopy Sciences, Hatfield, PA, USA) and were subsequently analyzed with conventional epifluorescence on a Nikon Eclipse 90i microscope and with a TCS SL Leica confocal system. Digital images were processed and assembled using the Adobe Photoshop software. No biased image manipulations were applied.

### Cuticle preparation of adult wings

Adult flies of the genotype *en2.4-Gal4*^*e22c*^*, UAS-flp/+; FRT*^*82B*^*/FRT*^*82B*^*, awd*^*j2A4*^ were collected. Wings were removed at the hinge, dehydrated in ethanol and mounted on microscope slides in lactic acid/ethanol (6:5). Wing images were captured by a Nikon Eclipse 90i microscope and acquired with a Nikon Digital Sight camera.

### Notch endocytosis assay

The assay was adopted from a published report [78] with modifications. Third instar larval wing discs were dissected in Schneider’s *Drosophila* medium (SDM) containing 1% fetal calf serum. The discs were cultured for 15 minutes on ice in the presence of the mouse monoclonal anti-NECD antibody. Excess antibody was rinsed away and the discs were incubated with fresh media at room temperature. The discs were dissected at different times and detected with anti mouse IgG.

### Co-localization and statistical analysis

Thresholds of confocal images were set in Adobe Photoshop to exclude background staining. Images were processed with the CDA plugin of ImageJ to obtain the Pearson’s coefficient. Statistical comparison was performed by two-tailed paired Student’s t-test (GraphPad Prism 6 software).

### Lysotracker staining

For Lysotracker *ex vivo* staining, females were dissected in SDM. Ovaries were collected, separated and incubated in medium containing 5 μM Lysotracker (DND-99, Molecular Probes) in soft agitation for five minutes at room temperature in the dark. Ovaries were then rapidly washed three times with fresh SDM, mounted and imaged immediately.

## Abbreviations

Avl: Avalanche; Awd: abnormal wing discs; BSA: bovine serum albumin; CSL: CBF1-Su(H)-Lag1; DSL: Delta/Serrate/Lag2; ESCRT: endosomal sorting complex required for transport; FasIII: Fasciclin III; FGFR: fibroblast growth factor receptor; GbeSu(H)m8: Grainyhead transcription factor binding site, Suppressor of Hairless binding sites, *Enhancer of split m8 gene*; GFP: green fluorescent protein; Hnt: Hindsight; Hrs: hepatocyte growth factor-regulated tyrosine kinase substrate; IgG: immunoglobulin G; MARCM: mosaic analysis with a repressible cell marker; MVBs: multivesicular bodies; NDPK: nucleoside diphosphate kinase; NECD: Notch extracellular domain; NEXT: Notch External Truncation; NICD: Notch intracellular domain; Nm23/NME: non metastatic cells; NRR: negative regulatory region; PBS: phosphate-buffered saline; PBT: Triton-100 in PBS; PDGF: platelet-derived growth factor; PVR: PDGF/VEGF receptor; Rab5CA: constitutively active Rab5; SDM: Schneider’s *Drosophila* medium; Shi: Shibire; UAS: upstream activating sequence; VEGF: vascular endothelial growth factor; YFP: yellow fluorescent protein.

## Competing interests

The authors declare that they have no competing interests.

## Authors' contributions

MI, MB, GN, JW and SD performed the experiments and participated in the discussion and conception part of the experiments. VC, GG and TH participated in the discussion, conceived and designed the experiments. VC and TH wrote the manuscript. All authors read and approved the final manuscript.

## Supplementary Material

Additional file 1: Figure S1 Notch signaling in wild type follicle cells is upregulated by either NICD or NEXT over-expression. Females of the genotype *hs-flp*, *UAS-mCD8GFP*/*act* > *CD2* > *Gal4*; *+*/*UAS-NICD* (A) or *hs-flp*, *UAS-mCD8GFP*/*act* > *CD2* > *Gal4*; *+*/*UAS-NEXT* (B) were dissected and the egg chambers were stained for Hnt (red). Over-expression of NICD at stage 7–8 in wild type follicle cells marked by GFP expression (green) enhances the level of Hnt expression in 51% of follicle cells (n = 100). Over-expression of NEXT at stage 7–8 in wild type follicle cells marked by the expression of GFP (green) enhances the level of Hnt expression in 92.5% of follicle cells (n = 40). Bars are 15 μm. Click here for file

Additional file 2: Figure S2 Presenilin expression pattern is not altered in *awd* mutant follicle cells. Polyclonal rabbit antibody against a C-terminal peptide in the putative hydrophilic loop region of Psn (anti-C-Psn) has been described [58]. Stage 6 and 7 egg chambers containing MARCM clones of *awd* mutant (marked by positive GFP expression) were stained for Psn (cyan) and NICD (red). Psn is ubiquitously expressed in intracellular punctates in both follicle cells and germ cells. No changes in either the expression level or the punctate pattern are observed in *awd* mutant cells. The egg chambers were dissected from *hs-flp/GbeSu(H)*_*m8*_*-lacZ; act-Gal4, UAS-GFP/+; FRT*^*82B*^*, act-Gal80/FRT*^*82B*^*, awd*^*J2A4*^ females. Bars are 5 μm. Click here for file

Additional file 3: Figure S3 Disrupted epithelial cells in *awd* mutant clone show abnormal Notch accumulation. Females of the genotype *en2.4-Gal4*^*e22c*^*, UAS-flp/+; FRT*^*82B*^*/FRT*^*82B*^*, awd*^*j2A4*^ were dissected and the egg chambers were stained for DNA (DAPI), Awd, NICD, and Avl as indicated. Abnormal Notch accumulation in large vesicles is observed in pile-up mutant epithelial cells (arrows), which co-localize with the early endosomal marker Avl (see also Additional file 5: Figure S5A,A’). Bar is 20 μm. Click here for file

Additional file 4: Figure S4 Small *awd* mutant clones exhibit loss of Hnt expression. Stage 7–8 egg chambers were dissected from *hs-flp/GbeSu(H)*_*m8*_*-lacZ; act-Gal4, UAS-GFP/+; FRT*^*82B*^*, act-Gal80/FRT*^*82B*^*, awd*^*J2A4*^ females and stained for Hnt (red) and DNA (cyan). Quantitative analysis of Hnt expression was perfomed in *awd* clones (GFP-positive cells, green) containing a maximum of 5 cells. In these small clones 93% of *awd* mutant cells lack Hnt expression (n = 42). Bar are 5 μm. Click here for file

Additional file 5: Figure S5 Analysis of Notch vesicle co-localization with Avl and Hrs. In *awd* mutant cells, Notch accumulates in Avl-positive and Hrs-negative early endosomes. Stage 7–8 egg chambers were dissected from *hs-flp/GbeSu(H)*_*m8*_*-lacZ; act-Gal4, UAS-GFP/+; FRT*^*82B*^*, act-Gal80/FRT*^*82B*^*, awd*^*j2A4*^ females and stained for NICD and Avl **(A,A’)** or NICD and Hrs **(B,B’)**. Co-localization was analyzed by using ImageJ. The Pearson's coefficient ranges from +1 = complete correlation to −1 = anti-correlation, with 0 = no correlation. The mean values (n = 4) of Pearson’s coefficients for NICD and Avl **(A)** and for NICD and Hrs **(B)** in *awd*^*+*^ and *awd* mutant cells were plotted together with standard deviations (error bars). Statistical significance was calculated using the two-tailed paired t-test (** = P <0.01; N.S. = No Significant). **(A’)** Co-localization image of NICD and Avl based on the analysis of *awd* mutant cells and neighboring *awd*^*+*^ cells showed in Figure 6A. **(B’)** Co-localization image of NICD and Hrs based on the analysis of *awd* mutant cells and neighboring *awd*^*+*^ cells. Click here for file

Additional file 6: Figure S6 Analysis of Notch vesicle co-localization with Rab7. In *awd* mutant cells, Notch does not accumulate in Rab7-positive endosomes. Stage 7–8 egg chambers were dissected from *hs-flp/GbeSu(H)*_*m8*_*-lacZ; act-Gal4, UAS-GFP/+; FRT*^*82B*^*, act-Gal80/FRT*^*82B*^*, awd*^*j2A4*^ females and stained for NICD and Rab7. Co-localization was analyzed by using ImageJ. The mean values (n = 6) of Pearson’s coefficients for NICD and Rab7 in *awd*^*+*^ and *awd* mutant cells were plotted together with standard deviations (error bars) **(A)**. Statistical significance was calculated using the two-tailed paired t-test (N.S. = Not Significant). **(A’)** Co-localization image of NICD and Rab7 based on the analysis of *awd* mutant cells and neighboring *awd*^*+*^ cells showed in Figure 6C. Click here for file

Additional file 7: Figure S7 *awd*^*+*^ and *awd* mutant cells show similar Lysotracker staining patterns. The egg chambers were dissected from *hs-flp/GbeSu(H)*_*m8*_*-lacZ; act-Gal4, UAS-GFP/+; FRT*^*82B*^*, act-Gal80/FRT*^*82B*^*, awd*^*J2A4*^ females and stained for Lysotracker. GFP expression identifies *awd* mutant clones. There is no difference in acidified endosomal compartments between *awd*^*+*^ and *awd* mutant cells. Bar is 5 μm. Click here for file

Additional file 8: Figure S8 Analysis of Notch vesicle co-localization with Rab11. In *awd* mutant cells, Notch does not accumulate in Rab11-positive endosomes. Stage 7–8 egg chambers were dissected from *hs-flp/GbeSu(H)*_*m8*_*-lacZ; act-Gal4, UAS-GFP/+; FRT*^*82B*^*, act-Gal80/FRT*^*82B*^*, awd*^*j2A4*^ females and stained for NICD and Rab11. Co-localization was analyzed by using ImageJ. The mean values (n = 4) of Pearson’s coefficients for NICD and Rab11 in *awd*^*+*^ and *awd* mutant cells were plotted together with standard deviations (error bars) (A). Statistical significance was calculated using the two-tailed paired t-test (N.S. = Not Significant). (A’) Co-localization image of NICD and Rab11 based on the analysis of *awd* mutant cells and neighboring *awd*^*+*^ cells showed in Figure 6D. Click here for file

Additional file 9: Figure S9 Analysis of Notch vesicle co-localization with Rab5^CA^ and Hrs. Stage 7–8 egg chambers were dissected from *hs-flp/GbeSu(H)*_*m8*_*-lacZ; UAS-YFP-Rab5*^*CA*^*/+; tub-Gal4, FRT*^*82B*^*, tub-Gal80/FRT*^*82B*^*, awd*^*j2A4*^ females and stained for NICD and Hrs. Quantitative analysis of Notch vesicle co-localization with Rab5^CA^ and Hrs was performed. In *awd* mutant cells 87.1% of Notch vesicles co-localizes with Rab5^CA^ and 31.45% co-localizes with Hrs (n = 124). Co-localization was analyzed also by using ImageJ. The mean values (n = 6) of Pearson’s coefficients for NICD and Rab5^CA^ (A) and for NICD and Hrs (B) in *awd* mutant cells are reported with standard deviations. (A) Co-localization image of NICD and Rab5^CA^ and (B) co-localization image of NICD and Hrs are based on the analysis of *awd* mutant cells showed in Figure 8C. Click here for file

Additional file 10 Supplementary experimental procedures. Click here for file
