# Supplementary figures and images for: Notch signaling during development requires the function of awd, the Drosophila homolog of human metastasis suppressor gene Nm23
Source: BMC Biol. 2014 Feb 14;12:12. doi: 10.1186/1741-7007-12-12 (PMC3937027; doi:10.1186/1741-7007-12-12)

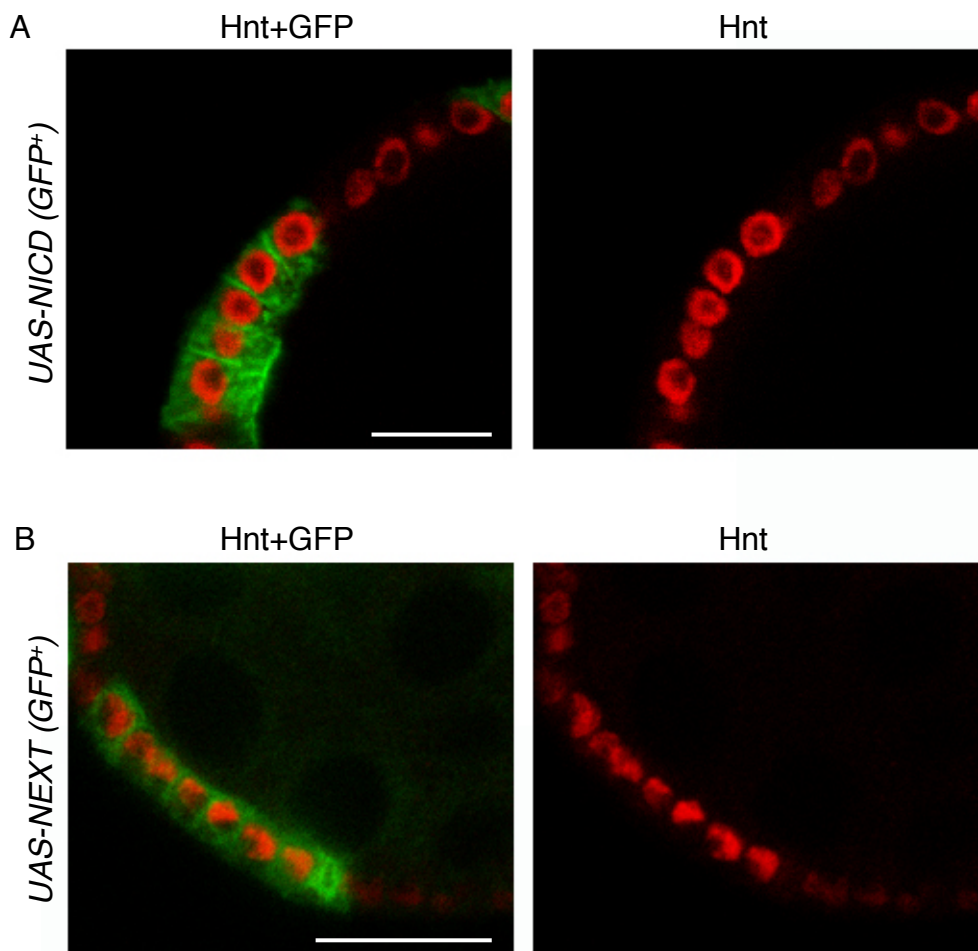

**Additional file 1 – Figure S1**

Supplement: Additional file 1: Figure S1 — Notch signaling in wild type follicle cells is upregulated by either NICD or NEXT over-expression. Females of the genotype hs-flp, UAS-mCD8GFP/act > CD2 > Gal4; +/UAS-NICD (A) or hs-flp, UAS-mCD8GFP/act > CD2 > Gal4; +/UAS-NEXT (B) were dissected and the egg chambers were stained for Hnt (red). Over-expression of NICD at stage 7–8 in wild type follicle cells marked by GFP expression (green) enhances the level of Hnt expression in 51% of follicle cells (n = 100). Over-expression of NEXT at stage 7–8 in wild type follicle cells marked by the expression of GFP (green) enhances the level of Hnt expression in 92.5% of follicle cells (n = 40). Bars are 15 μm. [file 1741-7007-12-12-S1.pdf]

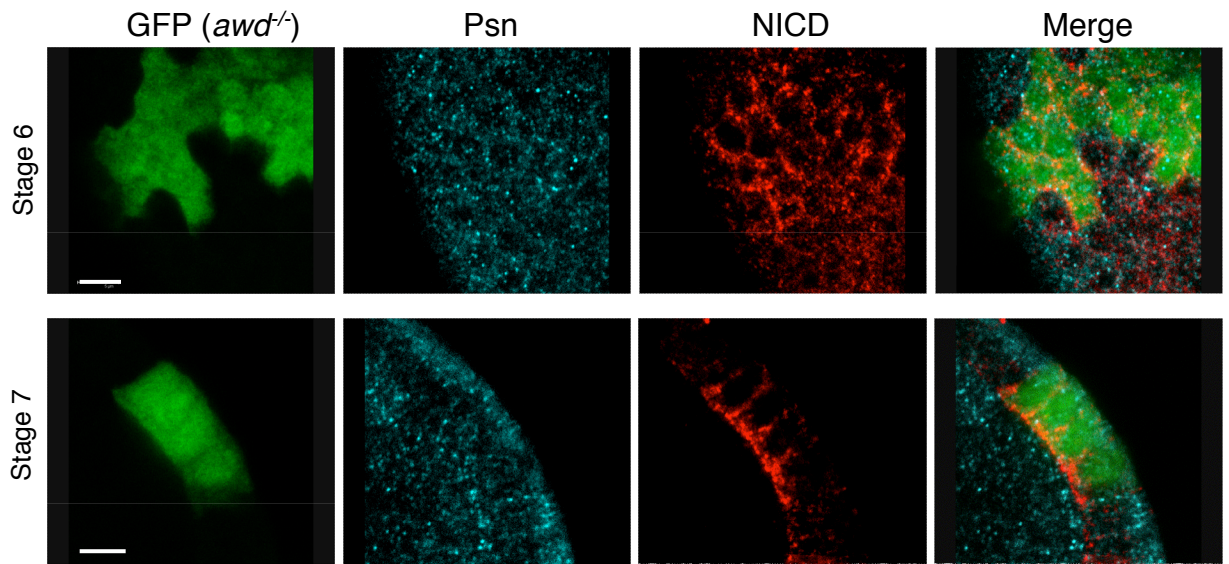

**Additional file 2 – Figure S2**

Supplement: Additional file 2: Figure S2 — Presenilin expression pattern is not altered in awd mutant follicle cells. Polyclonal rabbit antibody against a C-terminal peptide in the putative hydrophilic loop region of Psn (anti-C-Psn) has been described [58]. Stage 6 and 7 egg chambers containing MARCM clones of awd mutant (marked by positive GFP expression) were stained for Psn (cyan) and NICD (red). Psn is ubiquitously expressed in intracellular punctates in both follicle cells and germ cells. No changes in either the expression level or the punctate pattern are observed in awd mutant cells. The egg chambers were dissected from hs-flp/GbeSu(H) m8 -lacZ; act-Gal4, UAS-GFP/+; FRT 82B , act-Gal80/FRT 82B , awd J2A4 females. Bars are 5 μm. [file 1741-7007-12-12-S2.pdf]

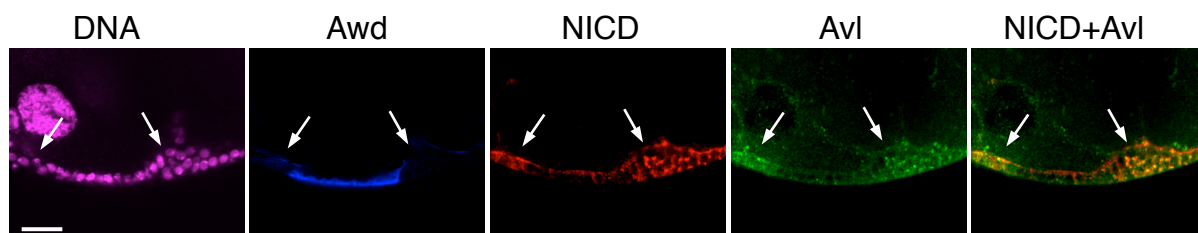

**Additional file 3 – Figure S3**

Supplement: Additional file 3: Figure S3 — Disrupted epithelial cells in awd mutant clone show abnormal Notch accumulation. Females of the genotype en2.4-Gal4 e22c , UAS-flp/+; FRT 82B /FRT 82B , awd j2A4 were dissected and the egg chambers were stained for DNA (DAPI), Awd, NICD, and Avl as indicated. Abnormal Notch accumulation in large vesicles is observed in pile-up mutant epithelial cells (arrows), which co-localize with the early endosomal marker Avl (see also Additional file 5: Figure S5A,A’). Bar is 20 μm. [file 1741-7007-12-12-S3.pdf]

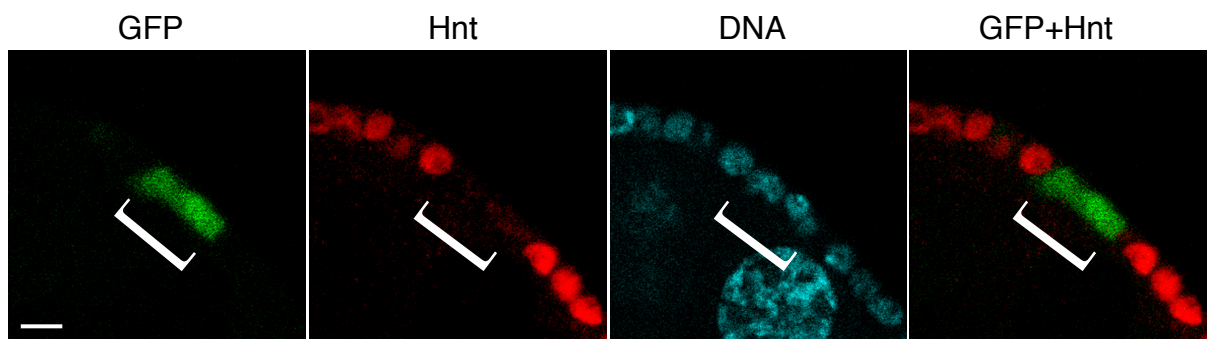

**Additional file 4 – Figure S4**

Supplement: Additional file 4: Figure S4 — Small awd mutant clones exhibit loss of Hnt expression. Stage 7–8 egg chambers were dissected from hs-flp/GbeSu(H) m8 -lacZ; act-Gal4, UAS-GFP/+; FRT 82B , act-Gal80/FRT 82B , awd J2A4 females and stained for Hnt (red) and DNA (cyan). Quantitative analysis of Hnt expression was perfomed in awd clones (GFP-positive cells, green) containing a maximum of 5 cells. In these small clones 93% of awd mutant cells lack Hnt expression (n = 42). Bar are 5 μm. [file 1741-7007-12-12-S4.pdf]

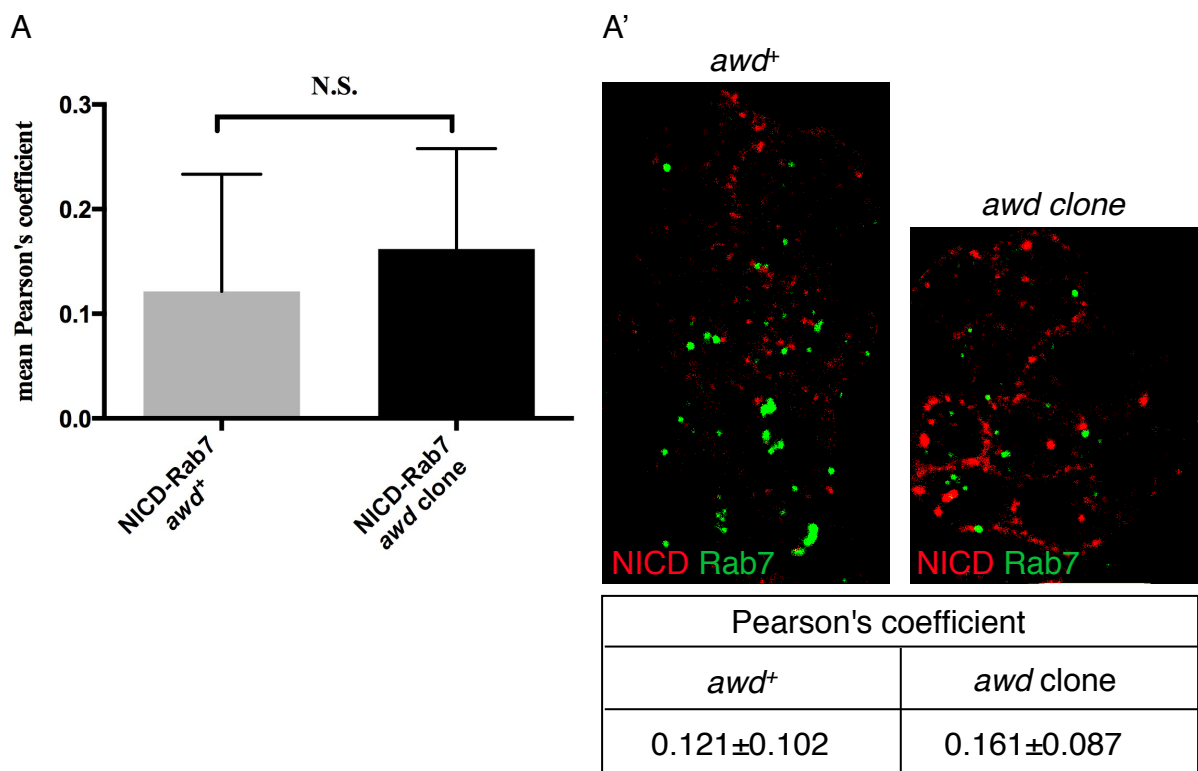

**Additional file 6 – Figure S6**

Supplement: Additional file 6: Figure S6 — Analysis of Notch vesicle co-localization with Rab7. In awd mutant cells, Notch does not accumulate in Rab7-positive endosomes. Stage 7–8 egg chambers were dissected from hs-flp/GbeSu(H) m8 -lacZ; act-Gal4, UAS-GFP/+; FRT 82B , act-Gal80/FRT 82B , awd j2A4 females and stained for NICD and Rab7. Co-localization was analyzed by using ImageJ. The mean values (n = 6) of Pearson’s coefficients for NICD and Rab7 in awd + and awd mutant cells were plotted together with standard deviations (error bars) (A). Statistical significance was calculated using the two-tailed paired t-test (N.S. = Not Significant). (A’) Co-localization image of NICD and Rab7 based on the analysis of awd mutant cells and neighboring awd + cells showed in Figure 6C. [file 1741-7007-12-12-S6.pdf]

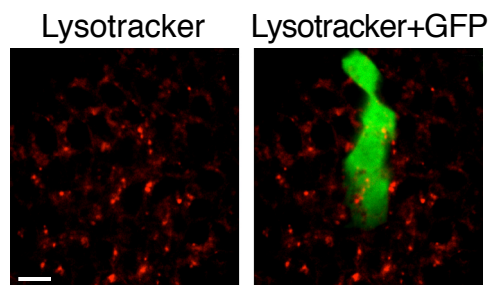

**Additional file 7 – Figure S7**

Supplement: Additional file 7: Figure S7 — awd + and awd mutant cells show similar Lysotracker staining patterns. The egg chambers were dissected from hs-flp/GbeSu(H) m8 -lacZ; act-Gal4, UAS-GFP/+; FRT 82B , act-Gal80/FRT 82B , awd J2A4 females and stained for Lysotracker. GFP expression identifies awd mutant clones. There is no difference in acidified endosomal compartments between awd + and awd mutant cells. Bar is 5 μm. [file 1741-7007-12-12-S7.pdf]

A

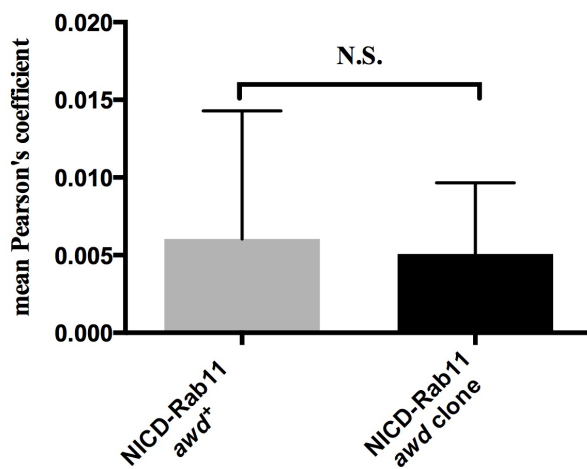

A'

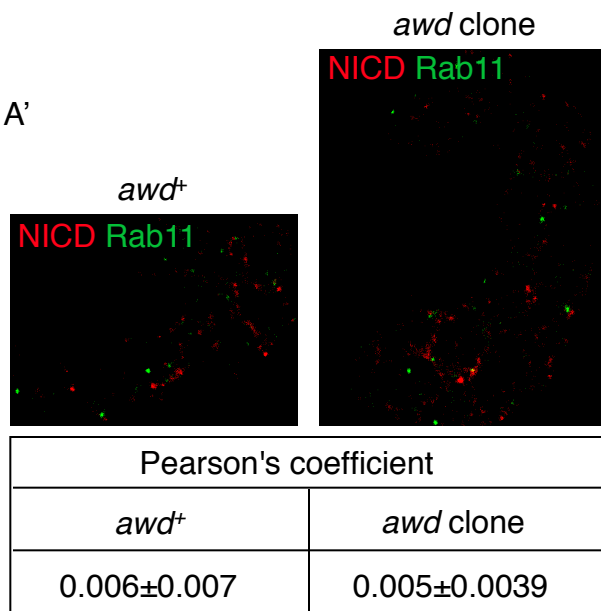

Additional file 8 – Figure S8

Supplement: Additional file 8: Figure S8 — Analysis of Notch vesicle co-localization with Rab11. In awd mutant cells, Notch does not accumulate in Rab11-positive endosomes. Stage 7–8 egg chambers were dissected from hs-flp/GbeSu(H) m8 -lacZ; act-Gal4, UAS-GFP/+; FRT 82B , act-Gal80/FRT 82B , awd j2A4 females and stained for NICD and Rab11. Co-localization was analyzed by using ImageJ. The mean values (n = 4) of Pearson’s coefficients for NICD and Rab11 in awd + and awd mutant cells were plotted together with standard deviations (error bars) (A). Statistical significance was calculated using the two-tailed paired t-test (N.S. = Not Significant). (A’) Co-localization image of NICD and Rab11 based on the analysis of awd mutant cells and neighboring awd + cells showed in Figure 6D. [file 1741-7007-12-12-S8.pdf]

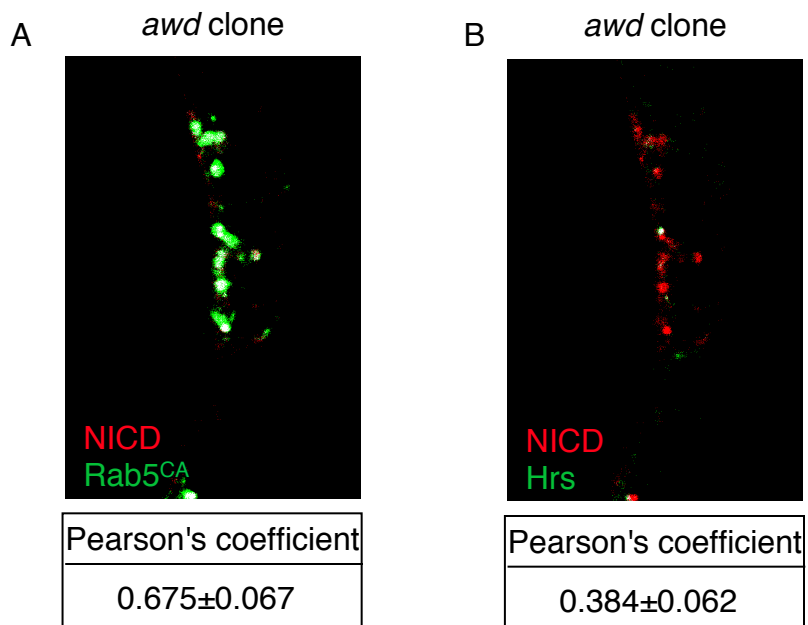

**Additional file 9 – Figure S9**

Supplement: Additional file 9: Figure S9 — Analysis of Notch vesicle co-localization with Rab5CA and Hrs. Stage 7–8 egg chambers were dissected from hs-flp/GbeSu(H) m8 -lacZ; UAS-YFP-Rab5 CA /+; tub-Gal4, FRT 82B , tub-Gal80/FRT 82B , awd j2A4 females and stained for NICD and Hrs. Quantitative analysis of Notch vesicle co-localization with Rab5CA and Hrs was performed. In awd mutant cells 87.1% of Notch vesicles co-localizes with Rab5CA and 31.45% co-localizes with Hrs (n = 124). Co-localization was analyzed also by using ImageJ. The mean values (n = 6) of Pearson’s coefficients for NICD and Rab5CA (A) and for NICD and Hrs (B) in awd mutant cells are reported with standard deviations. (A) Co-localization image of NICD and Rab5CA and (B) co-localization image of NICD and Hrs are based on the analysis of awd mutant cells showed in Figure 8C. [file 1741-7007-12-12-S9.pdf]
