# Supplementary material for: Notch signaling during development requires the function of awd, the Drosophila homolog of human metastasis suppressor gene Nm23
Source: BMC Biol. 2014 Feb 14;12:12. doi: 10.1186/1741-7007-12-12 (PMC3937027; doi:10.1186/1741-7007-12-12)
Supplement: Additional file 5: Figure S5 — Analysis of Notch vesicle co-localization with Avl and Hrs. In awd mutant cells, Notch accumulates in Avl-positive and Hrs-negative early endosomes. Stage 7–8 egg chambers were dissected from hs-flp/GbeSu(H) m8 -lacZ; act-Gal4, UAS-GFP/+; FRT 82B , act-Gal80/FRT 82B , awd j2A4 females and stained for NICD and Avl (A,A’) or NICD and Hrs (B,B’). Co-localization was analyzed by using ImageJ. The Pearson's coefficient ranges from +1 = complete correlation to −1 = anti-correlation, with 0 = no correlation. The mean values (n = 4) of Pearson’s coefficients for NICD and Avl (A) and for NICD and Hrs (B) in awd + and awd mutant cells were plotted together with standard deviations (error bars). Statistical significance was calculated using the two-tailed paired t-test (** = P <0.01; N.S. = No Significant). (A’) Co-localization image of NICD and Avl based on the analysis of awd mutant cells and neighboring awd + cells showed in Figure 6A. (B’) Co-localization image of NICD and Hrs based on the analysis of awd mutant cells and neighboring awd + cells. [file 1741-7007-12-12-S5.pdf]

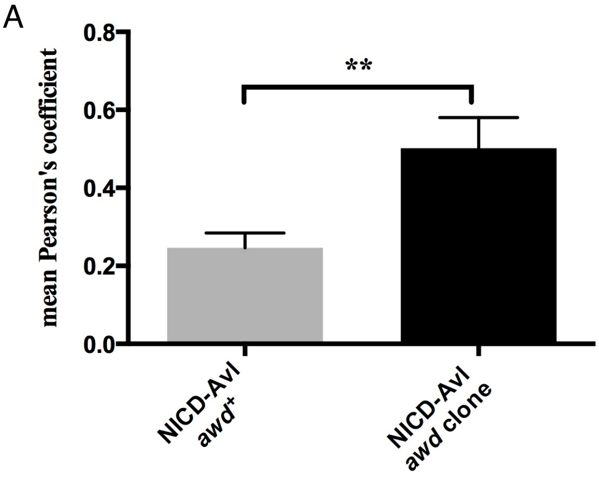

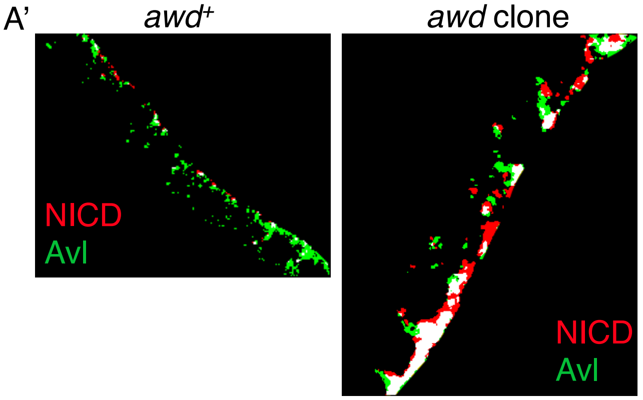

Pearson's coefficient	
awd+	*awd* clone
0.246±0.038	0.502±0.068

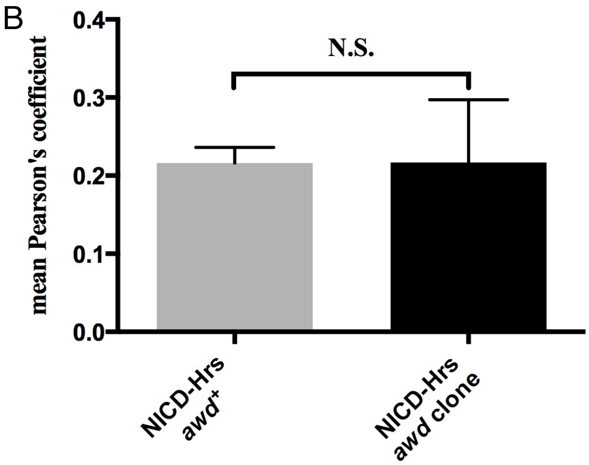

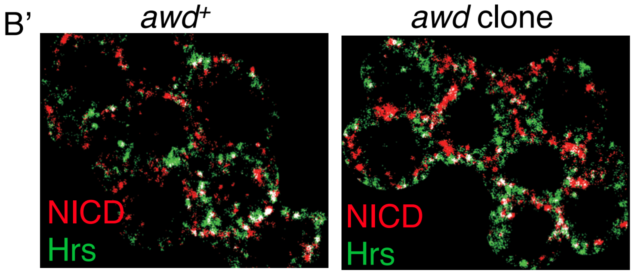

Pearson's coefficient	
awd+	*awd* clone
0.216±0.017	0.217±0.070

Additional file 5 – Figure S5
