# Supplementary material for: Notch signaling during development requires the function of awd, the Drosophila homolog of human metastasis suppressor gene Nm23
Source: BMC Biol. 2014 Feb 14;12:12. doi: 10.1186/1741-7007-12-12 (PMC3937027; doi:10.1186/1741-7007-12-12)
Supplement: Additional file 10 — Supplementary experimental procedures. [file 1741-7007-12-12-S10.pdf]

Supplementary experimental procedures

Genotypes of dissected females

1) *en2.4-GAL4*^{e22c}*, UAS-flp/+; FRT*^{82B}*/FRT*^{82B}*, awd*^{j2A4}

2) *en2.4-GAL4*^{e22c}*, UAS-flp/+; Ubi-GFP, FRT*^{82B}*/FRT*^{82B}*, awd*^{j2A4}

3) *en2.4-GAL4*^{e22c}*, UAS-flp/UAS-YFP-Rab5*^{wt}*; FRT*^{82B}*/FRT*^{82B}*, awd*^{j2A4}

4) *en2.4-GAL4*^{e22c}*, UAS-flp/UAS-YFP-Rab5*^{CA}*; FRT*^{82B}*/FRT*^{82B}*, awd*^{j2A4}

5) *hs-flp; +/+; Ubi-GFP, FRT*^{82B}*/FRT*^{82B}*, awd*^{j2A4}

6)*y, w, hs-flp/GbeSu(H)*$_{m8}$*-lacZ; UAS-YFP-Rab5*^{wt}*/+; Tub-Gal4, FRT*^{82B}*, Tub-Gal80/FRT*^{82B}*, awd*^{j2A4}

7) *y, w, hs-flp/GbeSu(H)*$_{m8}$*-lacZ; UAS-YFP-Rab5*^{CA}*/+; Tub-Gal4, FRT*^{82B}*, Tub-Gal80/FRT*^{82B}*, awd*^{j2A4}

8) *y, w, hs-flp/GbeSu(H)*$_{m8}$*-lacZ; UAS-YFP-Rab5*^{DN}*/+; Tub-Gal4, FRT*^{82B}*, Tub-Gal80/FRT*^{82B}*, awd*^{j2A4}

9) *y, w, hs-flp/GbeSu(H)*$_{m8}$*-lacZ; Act-Gal4, UAS-GFP/UAS-NICD; FRT*^{82B}*, Act-Gal80/FRT*^{82B}*, awd*^{j2A4}

10) *hs-flp/GbeSu(H)*$_{m8}$*-lacZ; Act-Gal4, UAS-GFP/UAS-NEXT; FRT*^{82B}*, Act-Gal80/FRT*^{82B}*, awd*^{j2A4}

11) shi^{2}/ shi^{2}

The crosses to obtain females of the genotype from 1) to 4) were performed at 18°C. Newly born females were transferred to fresh vials with *y, w*^{67c23} males and incubated at 29°C. Before dissection, the flies were transferred to fresh, yeasted food daily at 29°C for 5 days. The crosses to obtain females of the genotype from 5) to 10) were performed at 25°C. 1-2 days-old females were heat shocked three times for 1 hr at 37°C and then transferred to fresh vials with *y, w*^{67c23} males and incubated at 25°C. Before dissection, the flies were transferred to fresh, yeasted food daily at 25°C for 4 days.

1-2 days-old *shi*^{2}/*shi*^{2} females were transferred at 29°C for 6 hr and then dissected and processed for immunostaining

Genotypes of dissected larvae

1) *y, w, hs-flp; +/+; FRT*^{82B}*, Ubi-GFP/FRT*^{82B}*, awd*^{j2A4}

2) *y, w, hs-flp/GbeSu(H)*$_{m8}$*-lacZ; Act-Gal4, UAS-GFP/+; FRT*^{82B}*, Act-Gal80/FRT*^{82B}*, awd*^{j2A4}

Loss of function clones of *awd*^{j2A4} were induced at 48h after egg laying by 1 hr heat shock at 37°C.

Genotypes of flies for cuticle preparation of adult wings

1) *en2.4-GAL4*^{e22c}*, UAS-flp/+; FRT*^{82B}*/FRT*^{82B}*, awd*^{j2A4}
